# The first linkage map for Australo-Papuan Treefrogs (family: Pelodryadidae) reveals the sex-determination system of the Green-eyed Treefrog (*Litoria serrata*)

**DOI:** 10.1038/s41437-023-00642-5

**Published:** 2023-08-04

**Authors:** Lorenzo V. Bertola, Conrad J. Hoskin, David B. Jones, Kyall R. Zenger, Donald T. McKnight, Megan Higgie

**Affiliations:** 1 College of Science and Engineering, James Cook University, Townsville, QLD 4811 Australia; 2 Centre for Tropical Bioinformatics and Molecular Biology, James Cook University, Townsville, QLD 4811 Australia; 3 Centre for Sustainable Tropical Fisheries and Aquaculture, James Cook University, Townsville, QLD 4811 Australia; 4 Department of Environment and Genetics, School of Agriculture, Biomedicine and Environment, West Wodonga, La Trobe University, Melbourne, VIC 3690 Australia

**Keywords:** Genetic linkage study, Genome evolution

## Abstract

Amphibians represent a useful taxon to study the evolution of sex determination because of their highly variable sex-determination systems. However, the sex-determination system for many amphibian families remains unknown, in part because of a lack of genomic resources. Here, using an F1 family of Green-eyed Treefrogs (*Litoria serrata*), we produce the first genetic linkage map for any Australo-Papuan Treefrogs (family: Pelodryadidae). The resulting linkage map contains 8662 SNPs across 13 linkage groups. Using an independent set of sexed adults, we identify a small region in linkage group 6 matching an XY sex-determination system. These results suggest *Litoria serrata* possesses a male heterogametic system, with a candidate sex-determination locus on linkage group 6. Furthermore, this linkage map represents the first genomic resource for Australo-Papuan Treefrogs, an ecologically diverse family of over 220 species.

## Introduction

The mechanisms determining phenotypic sex vary greatly across the Tree of Life. In vertebrate animals, some species use genetic sex determination (GSD)—where sex is determined by a heritable genetic component, some use environmental sex determination—where sex is determined by the environmental conditions (e.g., temperature) experienced by the developing embryo, and some use both when the genetically determined sex is reversed later in life due to environmental cues (Capel 2017). Within GSD-only systems, a gradient in the genetic architecture of sex determination exists. At one end, sex is determined by segregating, heteromorphic (i.e., different size), non-recombining sex chromosomes that contain non-homologous genes. This is the case in many therian mammals, where the male possesses an XY pair of sex chromosomes of different size, with the male-determining master gene, *SRY*, being located on the shorter Y chromosome (Wallis et al. 2008). In birds, conversely, the female possesses heteromorphic ZW sex chromosomes, with sex being determined by the dosage of the *DMRT1* gene, located on the Z chromosome (Smith et al. 2009; Ioannidis et al. 2021). At the other end of the spectrum of GSD exist species with homomorphic (i.e., cytologically undifferentiated) recombining sex chromosomes containing homologous genes. One of the best-characterised examples is found in *Takifugu* puffer fish, where a single nucleotide polymorphism on the *Amrh2* gene on Chromosome 19 determines sex, with all heterozygous individuals developing as males while homozygous individuals develop as females (Ieda et al. 2018; Duan et al. 2021). Here we will refer to the homomorphic chromosomes harbouring the reference and alternate sex-determining alleles as the X and Y sex chromosomes in male heterogametic systems, respectively—and Z and W sex chromosomes in female heterogametic systems, following terminology established in the literature (Bachtrog et al. 2014; Capel 2017; Ma and Veltsos 2021).

All amphibians studied so far have GSD (Ma and Veltsos 2021). However, in contrast to the more conserved GSD systems in mammals and birds (although see Capel 2017), amphibians show a high diversity of GSD systems. These include homomorphic XY sex chromosomes (e.g., *Hyla* treefrogs; Dufresnes et al. 2021), heteromorphic ZW sex chromosomes (e.g., *Pseudis* foam frogs; Gatto et al. 2016); WO sex determination, where females possess an additional W chromosome (e.g., New Zealand *Leiopelma* frogs; Green 1988); both homo- and heteromorphic XY and ZW sex determination within the same species (e.g., Japanese *Glandirana rugosa*; Miura 2007); and species with multiple sets of sex chromosomes (e.g., Taiwanese frog *Odorrana swinhoana*; Miura et al. 2021). Importantly, the majority (~75%) of anuran amphibians (i.e., frogs and toads) assessed to date have homomorphic sex chromosomes (Eggert 2004; Ma and Veltsos 2021).

The study of sex determination in anurans (and amphibians more broadly) initially lagged behind other vertebrate groups because traditional cytogenetic methods could not identify homomorphic sex chromosomes, which are morphologically indistinguishable. Only recently, with the development of novel methods (e.g., Gamble and Zarkower 2014; Brelsford et al. 2017), additional studies have identified the sex-determination system and sex chromosomes in anuran species with homomorphic sex chromosomes (Brelsford et al. 2016a; Lambert et al. 2016; Jeffries et al. 2018; Sopniewski et al. 2019). Yet, despite the recent accumulating body of work on anuran sex determination, the sex-determining gene has been identified only in one species (*DM-W* gene in *Xenopus laevis*, Yoshimoto et al. 2008). A number of other studies have identified narrow sex-determining regions and/or sex-diagnostic markers, but not the sex-determining gene(s) (e.g., Brelsford et al. 2016b). Furthermore, work on sex determination in anurans has focused on a subset of genera and families, including *Xenopus* clawed frogs (family Pipidae) in Africa, *Rana* and *Pelophylax* true frogs (family Ranidae) in Eurasia and North America, *Hyla* treefrogs (family Hylidae) in Europe, *Eleutherodactylus* and *Pristimantis* rain frogs (family Hylidae) in Central and South America, *Engystomops* and *Gastrotheca* foam frogs (family Lepidodactylidae) in Central and South America, and *Leiopelma* frogs (family Leiopelmatidae) in New Zealand (reviewed in Ma and Veltsos 2021). Together, this body of work has documented the varied sex-determination systems present in anurans and highlighted the prevalence of both sex chromosome recombination (Guerrero et al. 2012; Dufresnes et al. 2015; Rodrigues et al. 2018) and turnover (i.e., the switch of the sex-determination role between different chromosome pairs) (Dufresnes et al. 2015; Jeffries et al. 2018). The diversity of sex-determination systems—and the many examples of young, recently evolved, recombining sex chromosomes—make anurans a useful group to understand the diversity and evolution of GSD and sex chromosomes. Yet, sex-determination data are lacking for entire anuran families, and hence our understanding of sex-determination diversity and evolution in anurans is currently limited.

Australo-Papuan Treefrogs (family: Pelodryadidae) are an ecologically and morphologically diverse group of treefrogs inhabiting Australia and Melanesia. They include more than 220 described species, with many of these species being listed as Endangered (Gillespie et al. 2020; Geyle et al. 2021), and many more species likely undescribed in Melanesia (Oliver et al. 2022). They represent a key group for a number of evolutionary questions, including speciation (e.g., Hoskin et al. 2005), adaptation to disease (e.g., Puschendorf et al. 2011; Banks et al. 2020; McKnight et al. 2019, 2020), and threatened species management (e.g., Beranek et al. 2021; West 2021). Like most anurans, no genome assembly currently exists for any species within this family, thus hampering evolutionary and conservation research in this group. Sex determination for this family has been investigated only in *Litoria aurea*, using ddRAD data and identifying 11 completely XY-linked markers, thus supporting an XY sex-determination system (Sopniewski et al. 2019). Yet, because of the lack of genome assemblies and linkage maps for any Pelodryadid Treefrog, the sex chromosome could not be identified.

In this study, we aimed to identify the sex-determination system and putative sex chromosome for the Green-eyed Treefrog (*Litoria serrata*) by answering the following questions: Does this species possess male (XY) or female (ZW) heterogametic sex-determination? And on which chromosome pair is the sex-determining locus located? We achieved this by producing the first linkage map for any Australo-Papuan Treefrog, using DArTseq data (Sansaloni et al. 2011; Kilian et al. 2012) and LepMap3 (Rastas 2017), from a single F1 family cross of two parents and more than 300 offspring. We then screened for sex markers in this species—for which the sex-determination system and sex-determining locus are unknown—and used permutation tests and the linkage maps to validate sex markers and identify the sex chromosome.

## Material and methods

### Reference mapping family and sample collection

To obtain a linkage mapping reference family, an amplexing pair of *L. serrata* was located at Henrietta Creek, in North-East Queensland, Australia, on 27 March 2014. The pair was temporarily placed in a container at the field site. The container was placed beside the creek and included stream water, rocks from the collection site, and a bubble aerator. Two rocks were placed against each other in the centre of the tank, with about 10 cm water depth. The aerator nozzle was placed between the two rocks to increase the oxygenation of water and to provide water flow through the rocks. This replicated shallow, flowing water in a rocky riffle zone, where this species breeds. The pair was then left overnight, and a clutch was laid between the rocks in the early hours of the morning. Tissue samples of the parents were collected with sterile surgical scissors to remove a single toe pad from toe II (i.e., second innermost) on the right foot. Toe pads were placed in 95% ethanol. The parents were then released at the site of capture. The egg clutch was left to develop within the container for 8 days until tadpoles reached Gosner stage 24/25. At this stage, 318 tadpoles were euthanised with an MS222 solution and placed in 95% ethanol. The remaining 150+ tadpoles were released into the stream at the site of the collection of the two adults. Sample collection and handling were conducted under James Cook University Ethics Approval (#A2123) and Queensland Department of Environment, Heritage, and Protection Scientific Purposes Permits (#WITK10437611/WISP10437711).

### Library preparation and sequencing

Genomic DNA extraction, library preparation and sequencing were conducted at Diversity Arrays Technology (DArT Pty Ltd) with the same methodology used before for *L. serrata* and *L. nannotis* (McKnight et al. 2019), *L. myola* (Bertola et al. 2023), and *L. dayi* (McKnight et al. 2020). This method uses a double-restriction digestion with the Pst1 and Sph1 restriction enzymes (RE). After digestion, proprietary DArT barcoded adapters between six and nine nucleotides long are then ligated to the RE site, and fragments are amplified through 30 PCR cycles. Equimolar amounts of PCR product are pooled, and the resulting library is sequenced (77 bp single-end reads) on an Illumina HiSeq 2500, producing an average of 1.25 million reads per individual.

To estimate genotyping error, 15% of samples were replicated by conducting library preparation twice from the same DNA extraction and thus sequencing each replicated sample in duplicate. Furthermore, the two linkage mapping parents were replicated three times to ensure higher coverage (~3 million reads per individual) and thus higher confidence in their genotypes, which is crucial for accurate linkage mapping.

To identify genetic markers linked and/or associated with phenotypic sex and use that information to determine the sex-determination system and identify the sex chromosome pair, we obtained raw fastq reads from McKnight et al. (2019) for 19 adult females and 19 adult males of *L. serrata*. All 38 sexed adults were collected from one population and were sampled within a 3 km radius in Girramay and Kirrama Range National Parks (McKnight et al. 2019). These samples were sequenced with the same protocol and at the same facility (i.e., DArT) as the current study. We could not use the tadpole offspring of the linkage mapping family herein because the tadpoles were euthanised before maturity and sex cannot be determined morphologically for tadpoles.

### De novo SNP identification and genotyping

Genotyping of the linkage mapping family and the sexed adults was conducted de novo using the software *stacks* v.2.55 (Rochette et al. 2019), as no genome assembly exists for any Australo-Papuan Treefrog. Raw fastq reads were trimmed to retain only high-quality reads for genotyping using the module *process_radtags* to remove reads with uncalled bases, discard reads with low-quality scores and rescue barcodes and cut sites (parameters -c, -q, and -r, respectively). The three separately generated fastq datasets for each of the two linkage mapping parents were concatenated to achieve higher coverage, while all other technical replicates were run separately at this stage.

*Stacks* parameters for building the catalogue of RADtags (where RADtag refers to a single assembled locus) were optimised using the r80 protocol following Paris et al. (2017). This process involves identifying the parameters that maximize the number of polymorphic RADtags found in 80% of the individuals in the study. The following parameters were tested, while retaining all other parameters as default: *M* values from 1 to 10 (i.e., the maximum distance allowed between two stacks), *m* values from 2 to 15 (i.e., the minimum depth of coverage required to create a stack) and *n* values from 1 to 5 (i.e., the number of mismatches allowed between RADtags built within individuals when building the catalogue from all individuals).

In addition, the software *Tiger* v.1.0 (Bresadola et al. 2020) was used to estimate the error rate between the independent technical replicates across the parameters tested in order to identify which parameters led to the lowest mismatch (i.e., lowest error rate). The input data for *Tiger* was obtained by running *stacks* with all combinations of parameters described above, and then retaining only bi-allelic markers present in at least half of all sequenced individuals and with a read depth between 5 and 50 using the *populations* module of *stacks*.

Finally, *stacks* was run de novo with the identified optimal parameters, running modules from *ustacks* to *gstacks* independently. This allowed us to build the marker catalogue using only the two linkage mapping parents as well as the 38 sexed adults from McKnight et al. (2019). Because the parents of a cross will contain all possible alleles present within the family, it is usually recommended to build the catalogue from the parents only, as including the progeny will add no new RADtags while possibly introducing additional genotyping errors (https://catchenlab.life.illinois.edu/stacks/manual/, accessed 17 October 2022).

### Linkage mapping

Markers to be used for linkage mapping (i.e., only for the two parents and the F1 offspring) were extracted from the overall catalogue using the *populations* module of *stacks* v.2.55. Only markers genotyped in at least 60% of individuals and with a minor allele frequency of 0.02 were retained. Individual genotype calls and markers with a read depth below 5 and above 50 were then filtered with *vcftools* v.0.1.16 (Danecek et al. 2011). More than one marker per RADtag was retained. Finally, individuals with more than 40% missing data were removed in R v.4.0.3 (R Core Team 2021) using custom scripts. At this stage, for individuals that had been sequenced twice as part of the technical replication, only one sample was retained by removing the sample with the most missing data from each pair.

An additional filtering step was conducted based on expected patterns of Mendelian segregation with custom R scripts (summarised in Supplementary Fig. S1). This workflow involved first assessing segregation distortion in the F1 offspring. Parent genotypes were then corrected if missing, and erroneous offspring genotypes silenced (i.e., converted to missing data). To do so, first, the observed genotype ratios in the offspring were compared with a chi-square *X*^2^ test to the genotype ratios expected under all seven possible Mendelian segregations (i.e., AAxAA, BBxBB, AAxBB, AAxAB, BBxAB, ABxAB, ABx--; where A and B represent the two alleles, and ABx-- represents a special case where one parent is heterozygous, one was not sequenced, and all offspring have either an AA or BB genotype in a 1:1 ratio). For the *X*^2^ test, a maximum of 2% erroneous genotypes (e.g., maximum of 2% BB offspring from an ABxAA cross) was allowed. Markers that matched no segregation at an alpha value of 0.005 (highly distorted markers) were removed. Markers assigned to a single segregation were retained if the parent genotypes matched the observed ratios in the offspring or if they could be matched by correcting only one parent with a missing genotype. All erroneous offspring genotypes were silenced, and uninformative markers (i.e., AAxAA, BBxBB, AAxBB) were discarded.

Linkage mapping was conducted using LepMap3 v.0.5 (Rastas 2017). The following modules were run in this order: *SeparateChromosomes2*, *JoinSingles2All* and *OrderMarkers2*. Linkage groups (LG) were identified with the *SeparateChromosomes2* module with a lodLimit of 40 and a sizeLimit of 20, followed by the *JoinSingles2All* module with a lodLimit of 10 and with iteration. Within each LG, markers were then ordered with the *OrderMarkers2* module. To obtain sex-specific maps, this module was run for either male-informative markers (informativeMask = 13) or female-informative markers (informativeMask = 23). The *OrderMarkers2* module was run 15 times for each LG for each sex-specific map, selecting the order with the highest likelihood. The resulting maps were inspected visually, and markers were removed if a single RADtag (which could include multiple markers) caused a gap of more than 10 centiMorgans (cM) at the end of a LG. RADtags were also removed if markers within a single RADtag occurred across multiple LGs, which is a biological impossibility and thus a sign of sequencing or genotyping errors. The *OrderMarkers2* module was then re-run 15 times for each LG for each sex-specific map, and once again, the run with the highest likelihood was selected. Sex-average maps were obtained by averaging the position for the double heterozygous markers occurring on both maps (i.e., (male position + female position)/2). The estimated genome coverage of the resulting linkage maps was calculated with the below equation, where *d* is the average spacing of markers, *n* is the number of markers, and *L* is the length of the linkage map (Bishop et al. 1983):

$$c=1-{e}^{-\tfrac{2{dn}}{L}}$$

### Identification of sex-linked and sex-associated markers

Two approaches were adopted to identify sex-linked and/or sex-specific single nucleotide polymorphisms. One approach assessed genotype proportions between sexes and compared them to the expectation under an XY (or ZW) sex-determination system to identify sex-linked markers, while the other approach used association tests between genotype and phenotypic sex to identify sex-associated markers. Note that we refer to the markers identified with the first approach as ‘sex-linked’, while the latter are referred to as ‘sex-associated’. Both approaches used the data collected from 19 adult males and 19 adult females (from McKnight et al. 2019). The two linkage map parents, which came from a different region to these 38 males and females, were not included in analyses of sex linkage because of their higher sequencing depth, and to reduce the noise from population structure. The dataset to identify sex markers was extracted from the *stacks* catalogue by retaining only markers with a minimum minor allele frequency of 0.05 using the *population* module of *stacks* v.2.55 (Rochette et al. 2019). In addition, only monomorphic or bi-allelic markers were retained using *vcftools* v.0.1.16 (Danecek et al. 2011). Monomorphic markers were retained as these could represent Y- or W-linked markers. No filter for maximum heterozygosity was applied as sex-linked markers are expected to have high heterozygosity (>0.75) in the heterogametic sex.

Sex-linked markers for both XY (male heterogametic) and ZW (female heterogametic) systems were identified following the analytical methodologies from Brelsford et al. (2017), Jeffries et al. (2018), and Lambert et al. (2016), who used ddRAD data in frogs to identify sex-linked markers. These methods search for Y- or W-linked markers either by using genotype frequency differences or presence/absence of markers between the two sexes. The rationale for methods based on genotype frequencies is that in a system with XY homomorphic sex chromosomes, in the sex-determining region markers linked to the sex-determining locus will have an excess of heterozygous calls in the males (which possess the Y allele) while females will be mostly homozygous. On the other hand, the rationale for presence/absence methods is that in highly differentiated sex-determining regions, the X and Y alleles will be so differentiated that they will be assembled as different RADtags, and thus the Y allele RADtag will be sequenced in the male only. In a ZW system, the opposite is true (i.e., females will have an excess of heterozygous calls and only females will possess the W allele). Because of the high rate of recombination between sex chromosomes in frogs, leading to poorly differentiated sex-determining regions between the two sex chromosomes, it was important to use analytical methods developed specifically for frogs that account for recombination between sex chromosomes by allowing non-perfect sex linkage (Dufresnes et al. 2015; Guerrero et al. 2012; Rodrigues et al. 2018). In total, we used four methods based on patterns of heterozygosity and one presence/absence method. The first three methods were developed by Brelsford et al. (2017). Method 1 looks for Y-linked SNPs (i.e., SNPs linked to the sex-determining locus in a male heterogametic system) by detecting SNPs where the X allele has a frequency higher than 0.95 in the females and between 0.4 and 0.6 in the males (i.e., an excess of heterozygotes in the males and a lack of heterozygotes in the females, allowing for imperfect linkage and genotyping errors). Method 2 looks for Y-linked SNPs by identifying markers where all females are homozygous and at least 50% of males are heterozygous. Method 3 looks for Y-linked markers by detecting RADtags absent in all females and present in at least 50% of males. Method 4, described in Lambert et al. (2016), tests for Y-linked SNPs by identifying markers where more than 75% of males are heterozygous, more than 80% of females are homozygous for the reference allele, fewer than 20% of females are heterozygous and fewer than 10% of females are homozygous for the Y-linked SNP. Our fifth method (Method 5), a modification of the Lambert et al. (2016) method, was used to search for Y-linked SNPs by identifying markers where more than 55% of males are heterozygous, more than 80% of females are homozygous for the reference allele, less than 20% of females are heterozygous and no females are homozygous for the Y-linked allele. In practice, Methods 4 and 5 are also looking for an excess of heterozygous genotypes in the males, but are less stringent than Methods 1 and 2 by allowing for a higher proportion of heterozygous and Y-linked homozygous genotypes in the females (Method 4), or a lower proportion of heterozygous males (Method 5). For all heterozygosity-based methods (all methods except for Method 3), only markers present in at least 60% of each sex were retained. To search for W-linked markers (to detect a ZW system), the expected genotype proportions were inversed for the two sexes for each of these five methods.

To assess whether the number of identified sex-linked markers was higher than expected by chance, permutation tests were run following Jeffries et al. (2018). A total of 10,000 permutations were run for each of the five methods by randomly assigning sex to the 38 sexed adults and assessing how many sex-linked markers were identified. To assess statistical significance, the number of identified sex-linked XY and ZW markers were then compared to the 95% and 99% quantiles obtained from the permutation tests.

With the same dataset, we also conducted tests of genotype-phenotype association to detect sex-associated SNPs. Because association tests are affected by fine-scale population structure, we first assessed population structure and relatedness within the data with the *gl.pcoa* and *gl.grm* functions from the *dartR* package v.2.0.4 (Mijangos et al. 2022), and removed any highly related clusters of individuals. We then conducted a genome-wide genotype-phenotype association using the *WGassociation* function of *SNPassoc* R package v.2.0-17 (González et al. 2007), using the codominant model, and retaining only bi-allelic markers genotyped for at least 80% of the individuals. We then identified markers significantly associated with sex by correcting p-values with the False Discovery Rate method within the *p.adjust* function of the *stats* base R package. Finally, the sex-linked and sex-associated markers identified were located on male, female, and sex-averaged linkage maps (if present), by matching their catalogue ID from *stacks*.

### Assessing synteny to available anuran resources

To assess synteny between the *L. serrata* linkage maps produced here and the *Xenopus tropicalis* chromosome level assembly, we conducted a stepwise blast approach similar to Jeffries et al. (2018). Using *blast* v.2.11.0 (Altschul et al. 1997), we first compared the consensus sequence for linkage map markers to the genome assemblies of five other hyloid anurans for which genome assemblies are publicly available (*Bufo bufo* GCA_905171765.1 (Streicher et al. 2021), *Bufo gargarizans* GCA_014858855.1 (Lu et al. 2021), *Leptodactylus fallax* GCA_947044405.1, *Platyplectrum ornatum* GCA_016617825.1 (Lamichhaney et al. 2021) and *Rhinella marina* GCA_900303285.1 (Edwards et al. 2018)). For each resulting hit, a 4-kb region was extracted and blasted to the *X. tropicalis* chromosome level genome assembly (GCA_000004195.4). At both stages, only hits with an e-value of 1E-15 and differing by more than four orders of magnitude from the second-best hit were retained. All assemblies were downloaded on 12 June 2023 from NCBI genome database (https://www.ncbi.nlm.nih.gov/genome/), with the exception of the *L. fallax* genome which was downloaded from the European Nucleotide Archive (https://www.ebi.ac.uk/ena/browser/view/GCA_947044405.1).

To assess synteny of the sex-linked and sex-associated markers identified here to known sex-determining loci in anurans, consensus sequences were extracted from the *stacks* catalogue and compared to the NCBI non-redundant nucleotide database (nt) and to the *L. aurea* reference and alternate sequences from Sopniewski et al. (2019), using the *Blastn* algorithm with *blast* v.2.11.0 (Altschul et al. 1997).

## Results

### Sequencing and de novo genotyping

After trimming and filtering raw reads with *process_radtags*, a total of 18.3 Gb were retained for the linkage mapping family (*n* = 389, including technical replicates) and 1.3 Gb were retained for the sexed adults (*n* = 38), respectively. The average number of bases was 47 million (stdev = 11, min = 22, max = 147) for the linkage mapping family, and 34 million (stdev = 7, min = 24, max = 59) for the sexed adults. For the two linkage mapping parents, which were replicated three times each, a total of 122 and 147 Mb of data were produced, respectively, for the male and female parent, compared to an average of 46 Mb for the offspring. An assessment of error rate between independent technical replicates using *Tiger* showed a high level of mismatch for heterozygous genotypes at read depths below 5×, with the error rate plateauing around 7–10× coverage (Supplementary Fig. S2). Thus, for all analyses, we removed markers with a read depth below 7×, which ensures an error rate for heterozygous calls below 3%, while retaining a good amount of data. Across all *stacks* parameters tested (as specified in the methods), a sharp increase in error rate was observed for *M* > 4 and *m* > 4 (Supplementary Fig. S3), and the number of markers present in at least 80% of samples plateaued around *M* = 4, *m* = 4, and *n* = 3 (Supplementary Fig. S4). Thus, to achieve a balance between accuracy and number of markers retained, the final parameters for *stacks* were *M* = 3, *m* = 4, and *n* = 3.

The resulting dataset included 24,042 RADtags, with an effective per sample mean coverage of 13× (stdev = 2.0×, min = 9×, max = 31×) for the linkage mapping family and of 11× (stdev = 1.5×, min = 10×, max = 16×) for the 38 sexed adults. The linkage mapping father and mother had the highest coverage, as expected, at 29× and 31×, respectively.

### Linkage mapping

The linkage mapping dataset contained 17,698 markers and 317 individuals, including the two parents. Three individuals were removed because of the low call rate. From the initial set of markers, 4652 were removed after filtering by segregation distortion, 867 were removed as they were uninformative, 2124 were removed because the parent genotype did not match the offspring segregation (Supplementary Table S1), 21 were not assigned to any LG, and 19 were removed as they caused long (>10 cM) gaps at the end of LGs. This included 7 markers from one RADtag occurring across two LGs. Details of the filtering steps can be found in Supplementary Table S2 and the online code.

The remaining 8662 markers were used for linkage mapping with LepMap3. For both sexes, we consistently recovered 13 LGs (Table 1). The resulting maps contained 5032 male-informative markers and 5277 female-informative markers, with a map length of 1380.72 and 1781.15 cM for the male and female map, respectively (Table 1 and Supplementary Figs. S5 and S6). The average female:male map length ratio was 1.29:1, ranging from 2:1 for the larger LGs to 1:1 for the smaller LGs, with LG9, LG11, and LG13 having a ratio less than 1:1. A high level of heterochiasmy was detected for all LGs, with the male map displaying clusters of markers near the centre of each LG with little recombination between them, while the markers in the female map were distributed more evenly along each LG (Supplementary Figs. S7 and S8). There were 1160 unique positions in the male map and 2161 in the female map, for an average non-zero interval (i.e., the average distance between unique positions) of 1.22 for the male map and 0.87 for the female map (Table 1).

**Table 1:** Linkage map summary metrics for the whole map and each linkage group (LG), for the sex-averaged (Avg), male (Male) and female (Fem) maps.

LG	Number of SNPs			Number of unique positions			Length (cM)			Non-zero intervals (cM)			F:M ratio
	Avg	Male	Fem	Avg	Male	Fem	Avg	Male	Fem	Avg	Male	Fem	
1	1118	636	696	447	94	284	211.55	106.76	212.20	0.47	1.15	0.75	1.99
2	1090	654	646	422	98	261	185.51	100.83	185.51	0.44	1.04	0.71	1.84
3	1075	636	644	372	74	231	181.57	110.07	182.89	0.49	1.51	0.80	1.66
4	942	569	562	388	103	233	167.43	111.36	168.42	0.43	1.09	0.73	1.51
5	771	410	491	325	111	177	133.31	105.22	133.31	0.41	0.96	0.76	1.27
6	741	428	450	303	83	182	163.16	108.13	163.81	0.54	1.32	0.91	1.52
7	542	315	340	250	85	144	111.77	102.72	114.08	0.45	1.22	0.80	1.11
8	471	276	293	224	88	125	117.53	106.62	120.52	0.53	1.23	0.97	1.13
9	462	254	291	241	82	136	115.96	115.96	111.34	0.48	1.43	0.82	0.96
10	447	282	251	230	101	112	108.37	108.69	108.21	0.47	1.09	0.97	1.00
11	356	197	220	182	76	93	91.88	94.21	81.11	0.51	1.26	0.88	0.86
12	340	169	230	182	71	99	103.70	103.68	103.70	0.57	1.48	1.06	1.00
13	307	206	163	178	94	84	105.16	106.47	96.04	0.59	1.14	1.16	0.90
MapAvg	666	387	406	288	89	166	138.22	106.21	137.01	0.49	1.22	0.87	1.29
Total	8662	5032	5277	3744	1160	2161	1796.90	1380.72	1781.15	–	–	–	–

Non-zero intervals refer to the average distance between unique positions, while F:M ratio is the ratio between map length of the female (F) versus male (M) map. MapAvg and Total represent the average and total values for the above metrics where relevant.

**Fig. 1:**
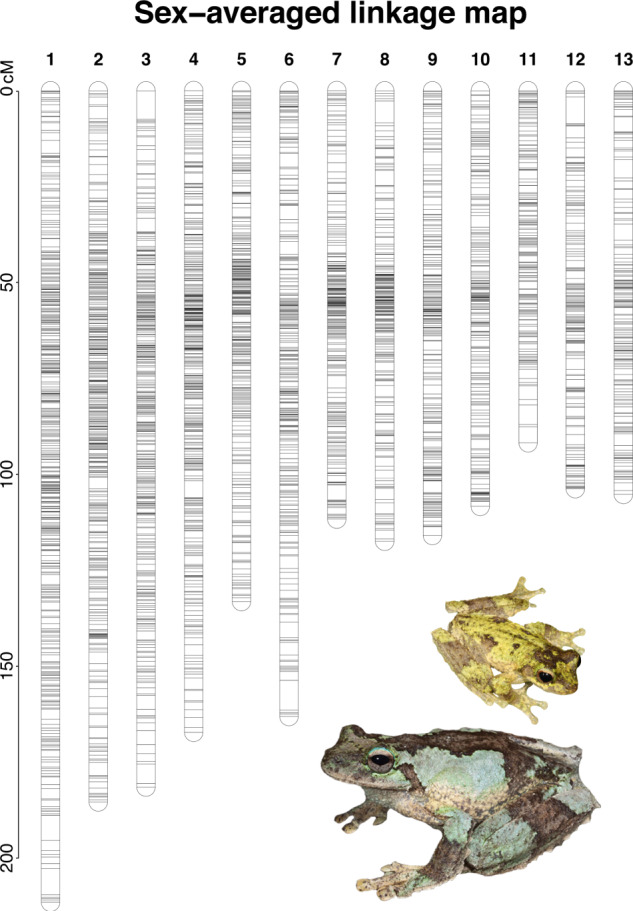
Sex-averaged linkage map for the Green-eyed Treefrog (*Litoria serrata*), showing linkage groups (LG) 1–13. Horizontal black lines indicate markers placed on the linkage map. The *y*-axis is in centiMorgans (cM). Figure produced with R package LinkageMapView and Adobe Photoshop. The photos show typical male (credit: Conrad Hoskin) and female (larger; credit: Anders Zimny) *L. serrata*.

A sex-average linkage map was obtained by averaging the position of double heterozygous markers (Fig. 1). The resulting map contained 8662 markers, across 3744 unique map positions and for an overall length of 1796.90 cM, with an average non-zero interval of 0.49 (Table 1).

### Identification of sex-linked markers

**Fig. 2:**
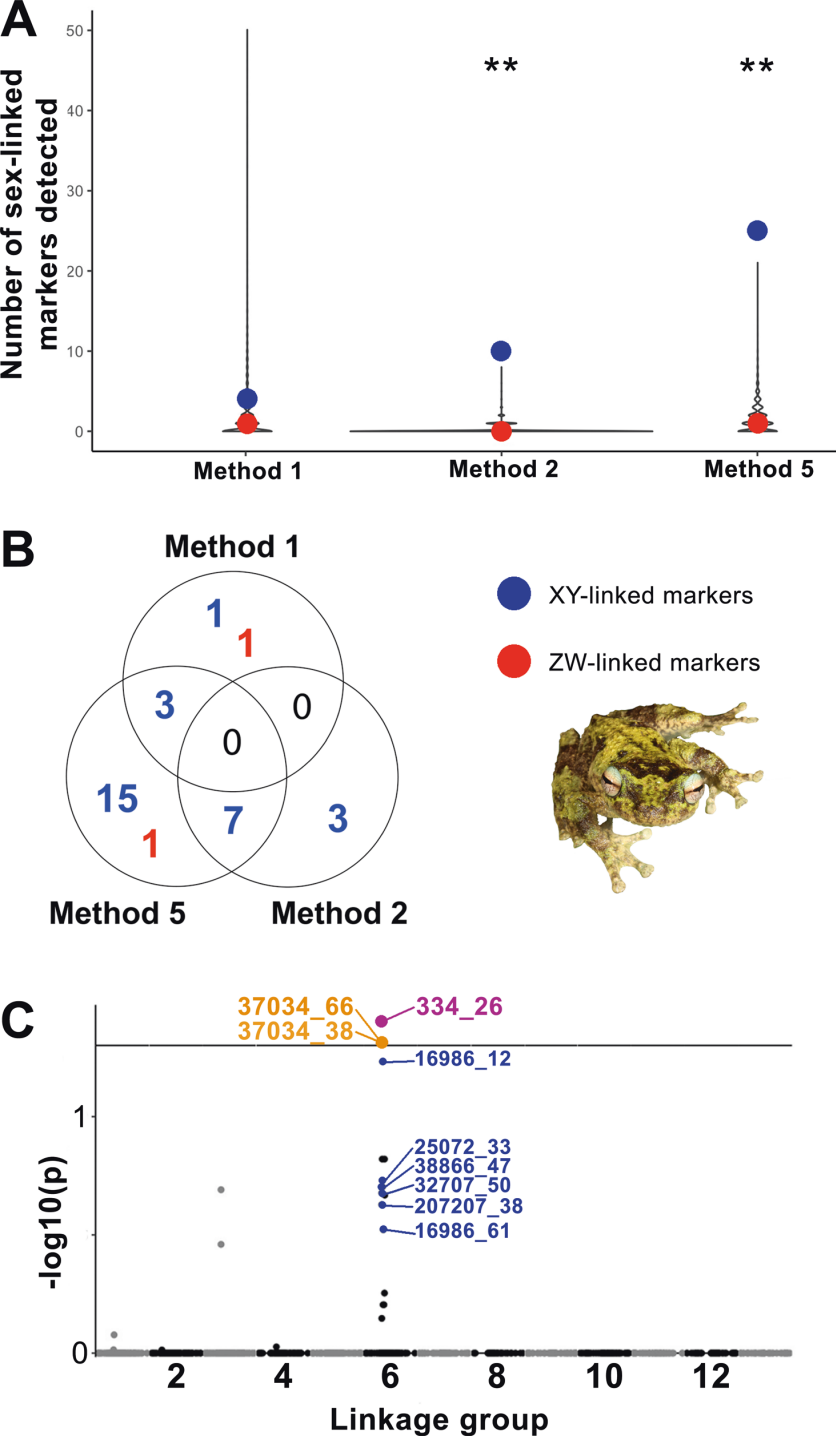
Summary of sex-linked and sex-associated markers detected. **A** Number of XY- and ZW-linked markers identified (blue and red dots, respectively) for Methods 1, 2, and 5, compared to the expected number of sex-linked markers detected by 10,000 permutations displayed as violin plots. Method 2 and 5 identified more XY-linked markers than expected by chance (*p* < 0.01). **B** Venn diagram showing the number of XY- and ZW-linked markers (blue and red numbers, respectively) identified by Methods 1, 2, and 5. The photo of the adult male *Litoria serrata* was provided by Conrad Hoskin. **C** Results from association tests, showing statistical significance on the *y*-axis as –log_10_(*p* value) versus position on the linkage map for all 13 linkage groups on the *x*-axis. Markers in purple and orange represent markers significantly associated with sex after FDR correction for multiple testing, with the colour depicting whether they were also identified by the sex-linkage methods (purple) or not (orange). Markers depicted in blue show all markers identified as XY-linked by sex-linkage approaches but not significantly associated with sex based on association approaches after FDR correction.

After filtering and retaining only monomorphic or bi-allelic markers, the dataset to identify sex markers contained 11,175 markers across 19 males and 19 females. A total of 31 putatively sex-linked markers were detected across the five sex-linkage methods, with 10 of these markers being identified by more than one method (Fig. 2 and Supplementary Table S3). Of the 31 unique sex-linked markers identified, 29 matched a male heterogametic system (i.e., XY-linked) and 2 matched a female heterogametic system (i.e., ZW-linked), from 25 unique RADtags. Method 1 identified four XY- and one ZW-linked markers; Method 2 identified 10 XY-linked markers; Method 3 and Method 4 identified no sex-linked markers; and Method 5 identified 39 XY and 1 ZW markers. Three of the XY markers identified with Method 1 and seven of the XY markers identified with Method 2 were also identified by Method 5. None of the identified sex-linked markers were completely sex-linked (Supplementary Table S4). Following Jeffries et al. (2018) Method 2 (Permutation Test: upper 99% quantile = 2, average = 0.1) and Method 5 (Permutation Test: upper 99% quantile = 8, average = 1.5) identified significantly more XY-linked markers than expected by chance (*p* < 0.01; Fig. 2 and Supplementary Figs. S9 and S10). Method 1 did not identify more XY-linked markers than expected by chance (Permutation Test: upper 99% quantile = 21, upper 95% quantile = 9, average = 2.1). The number of ZW-marker identified was not significant for both Method 1 and Method 5.

### Identification of sex-associated markers

Structuring and relatedness of individuals was assessed using the same dataset used to identify sex-linked markers. Six individuals (three males and three females) were removed as they clustered separately and showed higher-than-average relatedness. To conduct association tests, monomorphic markers and markers present in less than 80% of individuals were removed, leaving 9823 SNPs. A total of 14 sex-associated SNPs (across 10 unique RADtags) were detected (significantly associated with sex at an alpha value of 0.05 after FDR correction). Seven of these were also identified by sex-linkage methods. Importantly, these results did not change when including the related individuals. A qqplot of observed versus expected significance based on uniform distribution is available in Supplementary Fig. S11.

### Mapping of sex-linked and sex-associated markers

**Fig. 3:**
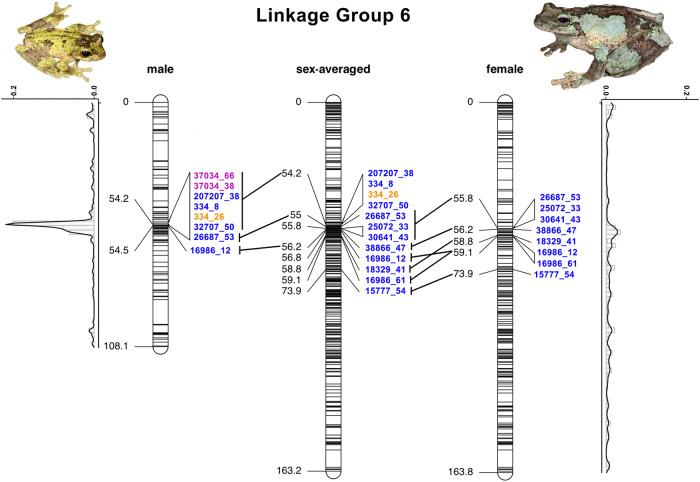
Position of all the XY-linked and sex-associated markers identified on LG6 for the male, sex-averaged, and female maps. Horizontal black lines indicate unique positions on the maps. Numbers in black on the left are in centiMorgans (cM) while numbers in blue, orange and purple represent the marker ID of markers identified through sex-linkage (blue), sex-association (orange) and both (purple) approaches. Thick diagonal lines connect synonymous markers across the maps. Density plots represent the marker density on the male (left) and female (right) LG6. Photos of a male (top left; credit: Conrad Hoskin) and female (top right; credit: Anders Zimny) adult *L. serrata*. Figure produced with R package LinkageMapView and Adobe Photoshop.

Of the 29 unique XY-linked markers, 13 were present in the linkage map, and all but one were located on LG6. The 12 XY-linked markers present on LG6 were located in a narrow region, between 54.2 and 54.5 cM on the male map, between 54.2 and 73.9 cM on the sex-averaged map, and between 55.8 and 73.9 cM on the female map (Fig. 3). Of the 14 sex-associated SNPs, only three occurred on the linkage maps, and all 3 occurred on LG6 at 54.17 cM on the male map (Fig. 3 and Supplementary Fig. S12). Only one of these markers was also identified by sex-linkage methods (334_26). Notably, the other two sex-associated markers not detected by sex-linkage methods had 100% heterozygosity in males and near 100% homozygosity in females, but a high missing rate (47%), hence why there were not identified by sex-linkage methods. Interestingly, the single XY-linked marker not located on LG6 was mapped to LG3, where a second, non-significant peak was detected with the sex-association method (Fig. 2C).

### Assessing synteny to available anuran resources

An average of 38 markers present on the *L. serrata* linkage maps returned a hit when compared to one of the five hyloid assemblies, with a minimum of 6 for the *P. ornatum* assembly and a maximum of 52 for the *L. fallax* assembly. When mapping to the *L. fallax*, *P. ornatum* and *R. marina* assemblies, LGs had a maximum of one match per scaffold. On the other hand, a moderate level of synteny was observed between *L. serrata* and the two *Bufo* species, with LGs LG1–6 and LG9–11 having between two and six matches to a chromosome level scaffold in the *B. bufo* assembly (Supplementary Table S5). Nevertheless, most *L. serrata* LGs matched more than one scaffold. Three markers from LG6 for instance mapped to the *B. bufo* assembly, one to scaffold SUPER_2 and two to scaffold SUPER_3 (Supplementary Table S5). After mapping to the *X. tropicalis* chromosome level assembly, a total of 21 unique matches were identified. LGs had between five (LG1) and zero (LG7, LG8, LG12 and LG13) unique matches to the *X. tropicalis* assembly, with three LGs only having one unique match (LG9–11). Two RADtags from LG6 mapped to *X. tropicalis* chromosomes (Chromosome 1 and 5, respectively).

Four RADtags returned matches from the Blastn search, with only four matches at an E-value below 1E-04, and no matches to known sex-determining genes (Supplementary Table S6). One RADtag, 69834, returned four matches, including to the DAPP1 and NT5C1A genes in *Bufo gargarizans*. These genes are located on chromosomes 1 and 5 of *X. tropicalis*, respectively. None of the sex markers identified in *L. serrata* matched those identified as sex markers in *L. aurea*. Details of the RADtag sequences, RADtag ID, marker ID, marker position on the linkage maps, parent and offspring genotypes from the F1 family, genotype counts and proportions from the sexed adults, and results from the sex-linkage and sex-association tests are available in Supplementary Table S4.

## Discussion

### Linkage map for Australo-Papuan Treefrogs

The linkage maps produced here for the Green-eyed Treefrog (*Litoria serrata*) represent the first linkage maps for any Australo-Papuan Treefrog (family: Pelodryadidae), a group of more than 220 species native to Australia and Melanesia. The linkage maps consistently recovered 13 LGs, representing 13 chromosomes and thus matching the known karyotype for *L. serrata* (Kakampuy et al. 2013) and other *Litoria* species (Mollard et al. 2018) from karyotyping analyses. Note that Kakampuy et al. (2013) referred to *L. serrata* as *L. genimaculata*, but their samples come from Australia, and Australian populations of ‘*L. genimaculata*’ were resurrected to *L. serrata* by Richards et al. (2010). A high degree of heterochiasmy was observed in the linkage maps, with heterogeneous recombination throughout the LGs in the female map and recombination cold spots around the centre of the LGs in the male map (Fig. 3 and Supplementary Fig. S7 and S8). Heterochiasmy has been widely documented in both ranid (Brelsford et al. 2016c; Jeffries et al. 2018) and hylid frogs (Brelsford et al. 2016a; Dufresnes et al. 2021) and is believed to be the result of male amphibians presenting only two terminal chiasmata during meiosis (reviewed in Perrin 2021).

Early linkage maps for amphibians mostly used microsatellite data and placed less than 100 markers (Kochan et al. 2003; Dufresnes et al. 2014; Rodrigues et al. 2018) with the exception of Wells et al. (2011), who produced a map for the model organism *Xenopus tropicalis* including 2886 simple sequence length polymorphism markers. Since then, linkage maps in frogs have improved substantially, and maps with thousands of markers are now available for *Hyla arborea* (Brelsford et al. 2016a), *Rana temporaria* (Jeffries et al. 2018), *Xenopus tropicalis* (Furman et al. 2020), and *Hyla sarda* and *Hyla savignyi* (Dufresnes et al. 2021). This study adds to this growing list by adding, for the first time, an Australo-Papuan Treefrog. The map presented here includes 8662 markers across 3744 unique positions, with an average non-zero interval of 0.47 cM. Only the *Rana temporaria* linkage map (Jeffries et al. 2018) successfully mapped more markers, and only an earlier *Xenopus tropicalis* map has a lower non-zero interval (Wells et al. 2011). However, it is important to note that some maps excluded markers heterozygous in both parents (e.g., Dufresnes et al. 2021), while others did not report the non-zero interval, which is likely to be smaller than the current study (e.g., Jeffries et al. 2018).

### Sex determination and sex chromosome in *Litoria serrata*

Thirty-one unique putative sex-linked markers were detected for *L. serrata*. Of these, 29 matched a male heterogametic (XY-linked) sex-determination system and only two matched a female heterogametic (ZW-linked) sex-determination system. Ten XY-linked markers were identified by more than one method, and none of the identified XY-linked markers was completely sex-linked (i.e., heterozygous females and/or homozygous males were detected for each marker). Method 2 and Method 5 identified more XY-linked markers than expected by chance (*p* < 0.01) after running permutation tests. Furthermore, thirteen of the XY markers were present on the linkage map produced here, and all but one mapped to a narrow region (<0.5 cM in the male map and <20 cM in the female map) in the centre of LG6 (Fig. 3). Association tests between the phenotypic sex and genome-wide markers identified 14 sex-associated markers after correcting for multiple testing. Seven of these were also identified by the sex-linkage methods, and, of these, three occurred on LG6 at 54.17 cM on the male linkage map (Fig. 3).

Our results thus suggest that a sex-determining locus for *L. serrata* is located on LG6, and that the species possesses an XY sex-determination system. This matches the sex-determination system of the only other Pelodryadid Treefrog for which it is known, the Green and Golden Bell Frog (*Litoria aurea*; Sopniewski et al. 2019). XY sex determination is also the prevalent sex-determination system in anurans (Tree of Sex Consortium 2014; Ma and Veltsos 2021), including Hylid Treefrogs (Dufresnes et al. 2015), with only *Hyla suweonensis* (Dufresnes et al. 2015), *H. sarda* and *H. savignyi* (Dufresnes et al. 2021), *Pseudis* foam frogs (Gatto et al. 2016), and a third of *Pristimantis* and *Eleutherodactylus* frogs possessing a ZW sex-determination system amongst all hylids studied thus far (reviewed in Ma and Veltsos 2021). The non-perfect sex linkage of the markers identified here and the lack of biologically significant results from the blast search suggest that none of these markers is directly involved in the sex-determination pathways. Notably, sex-linked and sex-associated markers detected here mostly fall within a region of low recombination in the centre of LG6 (Fig. 3). The low recombination in this region is likely what is preserving the linkage disequilibrium between the identified sex markers and the causal sex-determination locus. Without a genome assembly for this genus, it is not possible to assess how large this region is, what genes it contains, and what genes might be contributing to sex determination in *Litoria* frogs. A second region weakly associated with phenotypic sex was detected on LG3, where the only XY-linked marker not placed on LG6 occurred, and where a second, non-significant, peak was detected with the association tests (Fig. 2C). The XY-linked marker on LG3 was identified by Method 5, for which on average one false positive is to be expected based on the permutation tests. Thus, given the data presented here, it remains unclear whether the signal on LG3 represents a true association with phenotypic sex.

### Synteny to available anuran resources

Only 21 RADtags from the *L. serrata* linkage map could be mapped to the *X. tropicalis* assembly. Many LGs only had one (LG9–11) or no (LG7–8, LG12–13) RADtags mapped to an *X. tropicalis* chromosome, while four LGs (LG1–3, LG6) mapped to more than one *X. tropicalis* chromosome. More RADtags could be mapped to the intermediate hyloid genome assemblies. Nevertheless, because of the low overall number of matches, the high number of scaffolds, and LGs mapping to more than one scaffold, the level of synteny between *L. serrata* and the five hyloid species remains unclear. Furthermore, the sex markers identified in this study did not match any of the *L. aurea* sex-linked markers, nor any sex-determination genes in the NCBI non-redundant nucleotide database.

While none of the sex markers identified here mapped to known sex-determining genes, one of the sex-linked RADtags mapped to the DAPP1 gene, which is found on the *X. tropicalis* chromosome 1. Previous work has shown that a number of sex-linked markers from diverse anuran genera map to this chromosome (Miura 2017), including *Bufo*, *Hyla* and *Rana* (Brelsford et al. 2013). These results thus suggest the possibility that the sex-determining locus on LG6 of *L. serrata* might be homologous to the *X. tropicalis* chromosome 1 region, which has been co-opted for sex determination by a number of anurans (Brelsford et al. 2013; Miura 2017).

At this stage, it is thus not possible to confidently assess synteny between the genome of *L. serrata* and the chromosome level assembly of *X. tropicalis*. The low number of mapped markers is likely due to the short sequence length of the RADtags (~69 bp) and the high divergence between *Litoria* and the closest relative for which a genome assembly is available. Other studies that successfully matched linkage map markers to available frog genomes usually possessed at least a draft assembly from the same genus (e.g., Brelsford et al. 2017). It thus remains unknown whether LG6 in *L. serrata* matches known sex-determination chromosomes in anurans, and whether sex-determining loci in other Pelodryadid Treefrogs studied so far occur on the same chromosome.

### Conclusion and future directions

This study presents the first linkage map for any Australo-Papuan Treefrog (family: Pelodryadydae) and suggests that *L. serrata* possesses an XY sex-determination system. These results provide strong evidence for a sex-determining locus being present on LG6 between 54.2 and 73.9 cM, which thus represents a good candidate region to identify the sex-determining gene(s) in *Litoria*. Future work should further assess synteny between this linkage map and amphibian genomes, and further investigate the narrow candidate sex-determining region on LG6 to identify the sex-determining gene(s). Importantly, given the small extent of the identified sex-determining region on LG6, whole-genome sequencing (WGS) of additional sexed individuals could be used to provide a list of candidate sex-determining genes for further investigation. To this end, it will be crucial to produce a genome assembly for Australo-Papuan Treefrogs, to facilitate WGS studies and increase the mappability of the linkage map markers to other anuran genomic resources.

The linkage map for *L. serrata* presented here is a significant resource for studying Australo-Papuan Treefrogs. It will enable increased accuracy and power when estimating a number of relevant evolutionary and conservation-related metrics like effective population size and introgression, provide context when aiming to identify specific genomic regions as demonstrated here, and facilitate genome assemblies. This linkage map will thus provide a key resource to enable and bolster the study of Australo-Papuan Treefrogs.

### Supplementary information

Supplementary material

Supplementary Table S4

## Data Availability

Raw data from the Illumina sequencing were deposited to NCBI short read archive database (BioProject: PRJNA912233; BioSamples: SAMN32257171–SAMN32257597).

## Code availability

The code used for all analyses in this manuscript and for the production of figures, from the processing of fastq files to the assessment of synteny, as well as detailed summaries of software and R package versions, is available online as a static GitHub pages website at https://mboitui.github.io/HighDensityLinkageMap/.
