# Supplementary material for: The first linkage map for Australo-Papuan Treefrogs (family: Pelodryadidae) reveals the sex-determination system of the Green-eyed Treefrog (Litoria serrata)
Source: Heredity (Edinb). 2023 Aug 4;131(4):263–72. doi: 10.1038/s41437-023-00642-5 (PMC10539516; doi:10.1038/s41437-023-00642-5)

**Supplementary Material for:**

The first linkage map for Australo-Papuan Treefrogs (family: Pelodryadidae) reveals the sex determination system of the Green-eyed Treefrog (*Litoria serrata*).

Lorenzo V. Bertola^1,2*^, Conrad J. Hoskin^1^, David B. Jones^1,3^, Kyall R. Zenger^1,3^, Donald McKnight^1,4^, Megan Higgie^1,2^

^1^*James Cook University, College of Science and Engineering, Townsville, QLD 4811, Australia*

^2^*James Cook University, Centre for Tropical Bioinformatics and Molecular Biology, Townsville, QLD 4811, Australia*

^3^*James Cook University, Centre for Sustainable Tropical Fisheries and Aquaculture, Townsville, QLD 4811, Australia*

*^4^La Trobe University, Department of Environment and Genetics, School of Agriculture, Biomedicine and Environment Wodonga, VIC 3690, Australia*

*Corresponding author email: [Lorenzo.bertola@my.jcu.edu.au](mailto:Lorenzo.bertola@my.jcu.edu.au)

ORCID IDs: 0000-0002-1927-308X (LVB)

0000-0001-8116-6085 (CJH)

0000-0002-5862-6335 (DBJ)

0000-0001-8543-098X (DTM)

0000-0001-6817-8786 (KRZ)

0000-0002-2397-0240 (MH)

**Supplementary material 1**

**Mendelian filtering pipeline**

This section provides a visual representation of the custom pipeline for filtering markers used for linkage mapping based on expected patterns of Mendelian inheritance.


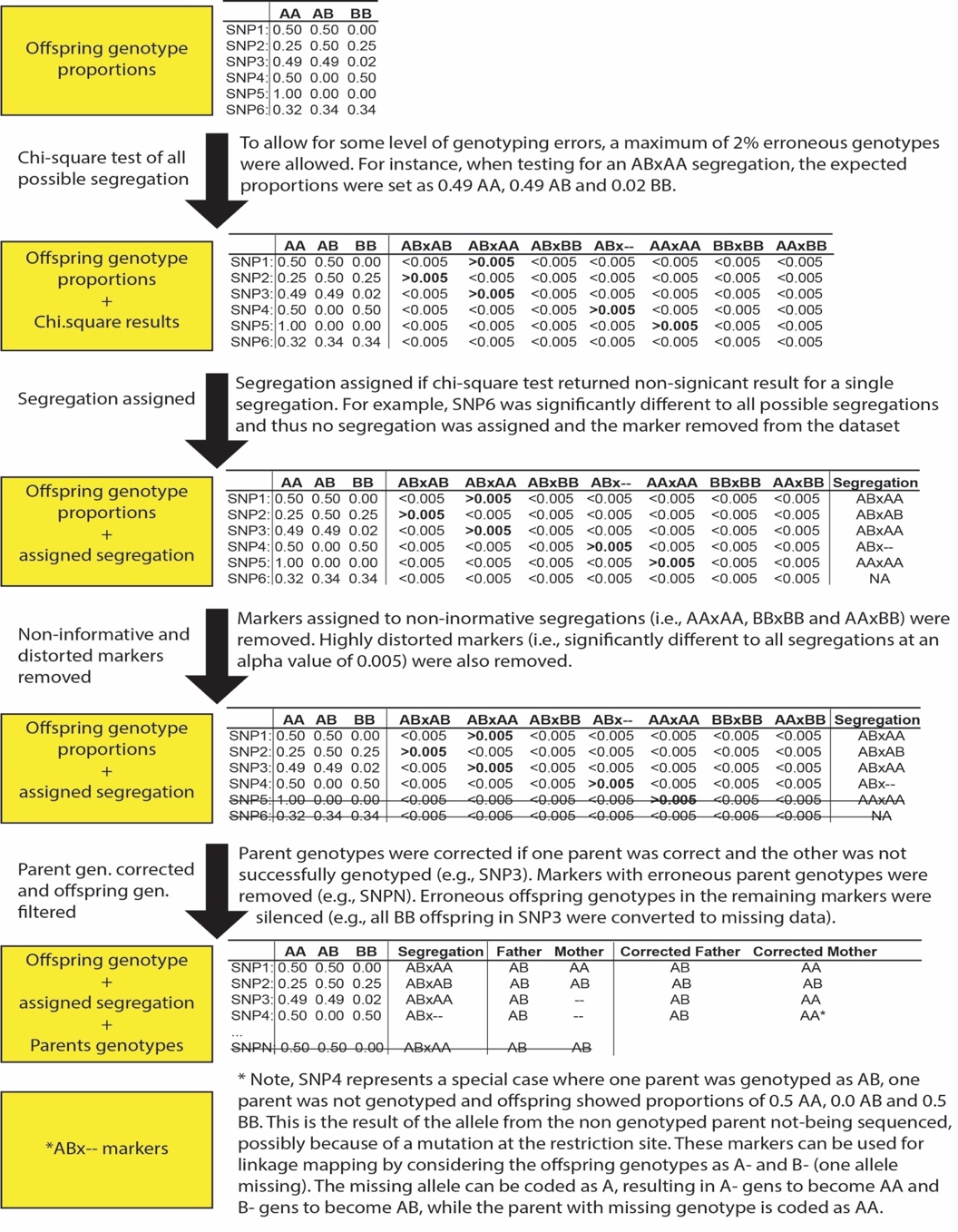


**Figure S1**: Visual summary of mendelian segregation filtering pipeline adopted. Yellow boxes denote the data being processed, while the text next to the black arrow denotes the actions being take. Bold text highlights parameters influencing the handling of data, while strikethrough text highlights markers being removed from the dataset.

**Supplementary material 2**

**Optimizing stacks parameters**

This section reports the different metrics used to select the set of stacks parameters (i.e., M – the maximum distance allowed between two stacks; m – the minimum depth of coverage required to create a stack; and n – the number of mismatches allowed between RADtags built within individuals when building the catalogue from all individuals), as well as the read depth filtering threshold (Fig. S2).


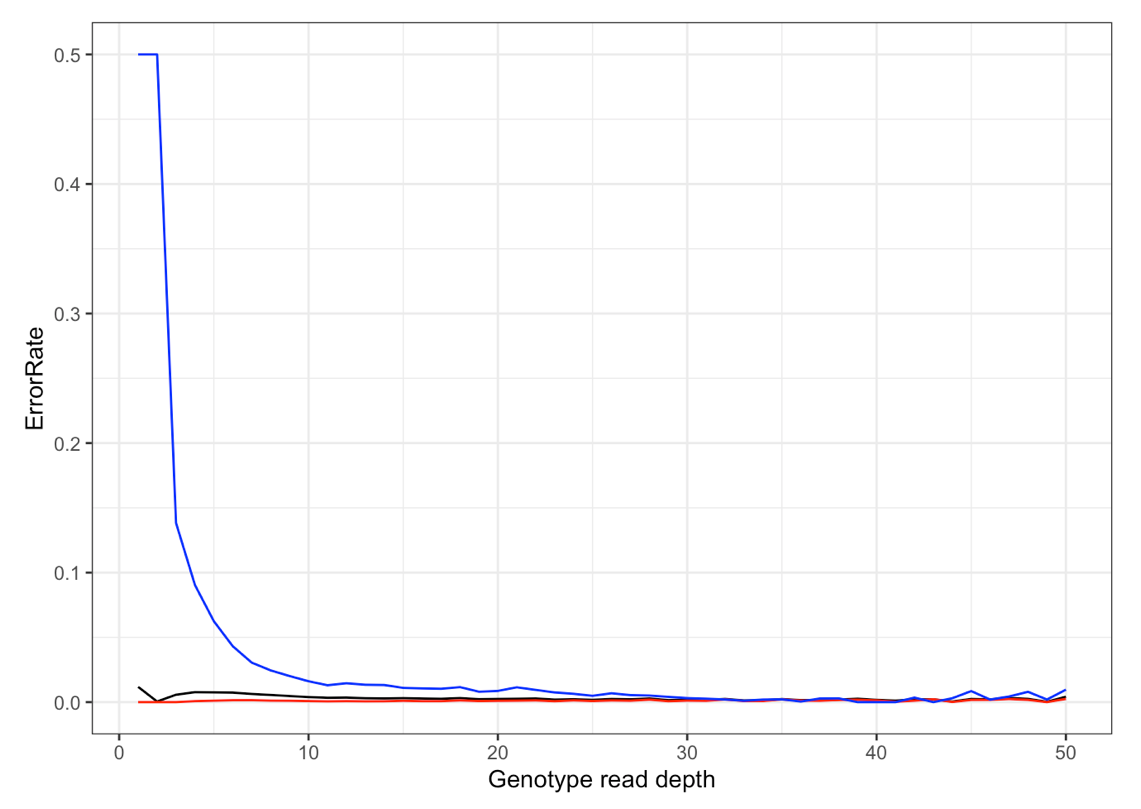


**Figure S2**: Error rate (mismatch between technical replicates) at different read depths for heterozygous (blue), all (black) and homozygous (red) calls.


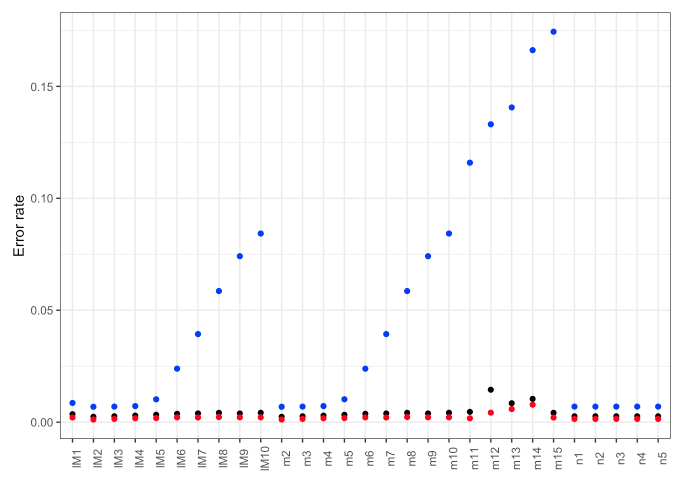


**Figure S3**: Error rate (mismatch between technical replicates) at different stacks parameters for heterozygous (blue), all (black) and homozygous (red) calls, for parameters M, m and n. Note that n doesn’t change as error rate is estimated from individual replicates, while n is involved in the merging of loci into the catalogue after stacks have been assembled in the individuals.


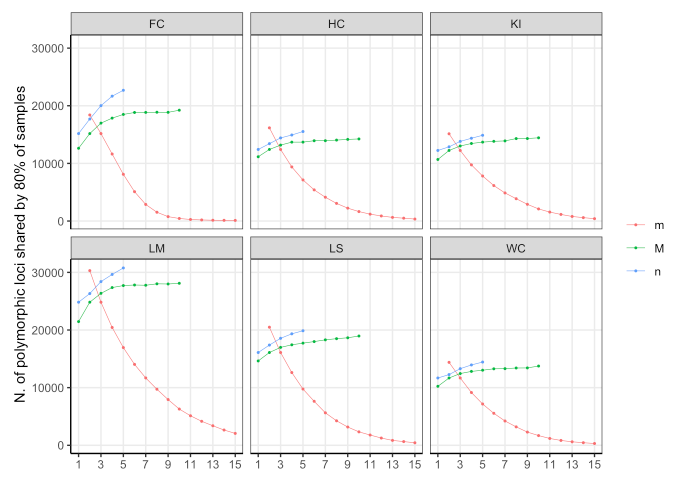


**Figure S4**: Number of polymorphic loci shared by 80% of samples across values of stack parameters, for the linkage mapping family (HC), the adult sexed individuals (LS), three additional linkage mapping families not presented in this study (FC, KI, WC) and an additional population genomic dataset for a sister species (LM: Litoria myola).

**Supplementary material 3**

**Filtering of linkage map data**

Details of filtering steps for the linkage mapping data, including the filtering threshold adopted for each step, the starting number of markers, the number of markers removed and the number of markers retained.

**Table S2**: Summary of filtering steps for markers used for linkage mapping.

| **Filter** | **Threshold** | **Starting** | **Removed** | **Retained** |
| --- | --- | --- | --- | --- |
| Call Rate | 0.6 | 17698 | 1353 | 16345 |
| Segregation distortion | 0.005 | 16345 | 4652 | 11693 |
| Uninformative markers | NA | 11693 | 867 | 10826 |
| Wrong parent genotype | NA | 10826 | 2124 | 8702 |
| Not assigned to LG | see text | 8702 | 21 | 8681 |
| Markers causing long gaps | > 10 cM gap | 8681 | 19 | 8662 |

**Supplementary material 4**

**Linkage map summary figures**

Visual summary of the sex-specific linkage maps, showing the male and female linkage maps (Fig. S5 and Fig. S6 respectively) and patterns of heterochiasmy (Fig. S7-8).

**Figure S5**: Male linkage map. Horizontal black lines represent unique map positions. Y axis is in centiMorgans (cM). Figure produced with R package LinkageMapView.

**Figure S6**: Female linkage map. Horizontal black lines represent unique map positions. Y axis is in centiMorgans (cM). Figure produced with R package LinkageMapView.


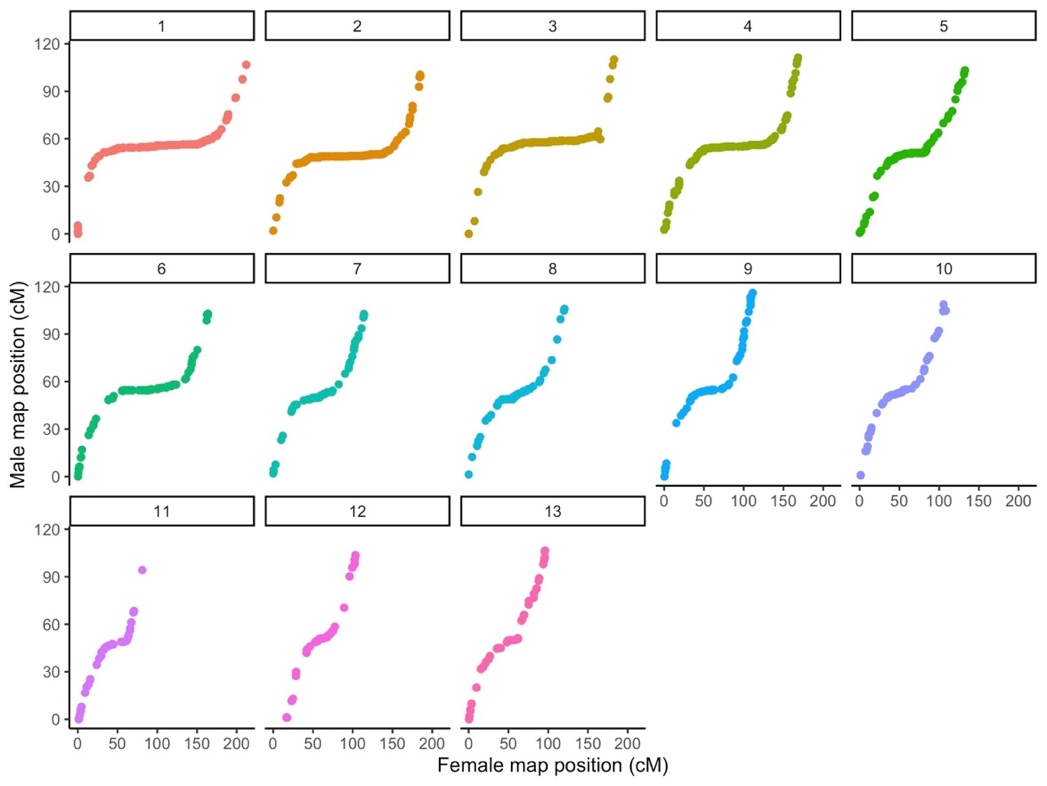


**Figure S7**: Comparison of male and female map position (in centiMorgans) for double heterozygous markers for the 13 linkage groups
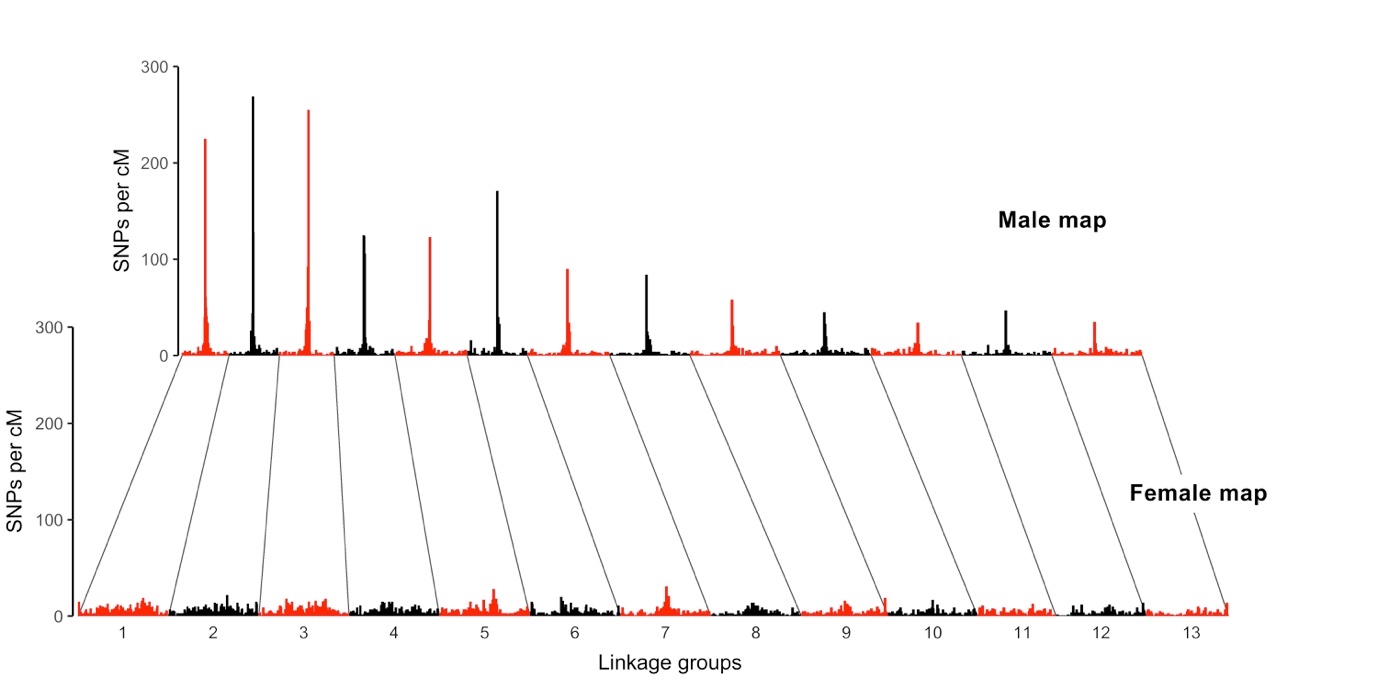


**Figure S8**: Marker density depicted as SNPs per centimorgan (cM) in the male (top) and female (bottom) linkage maps, displayed for each of the 13 linkage groups.

**Supplementary material 5**

**Details of sex-linked markers and permutation tests**

Details on the number of XY- and ZW- linked markers detected by each of the five methods, and visual representation of the permutation test results.

**Table S3**: Number of XY- and ZW- linked markers identified by the five methods.

| **Method** | **XY** | **ZW** | **Total** |
| --- | --- | --- | --- |
| 1 | 4 | 1 | 5 |
| 2 | 10 | 0 | 10 |
| 3 | 0 | 0 | 0 |
| 4 | 0 | 0 | 0 |
| 5 | 25 | 1 | 26 |
| Total | 39 | 2 | 41 |
| Unique | 29 | 2 | 31 |


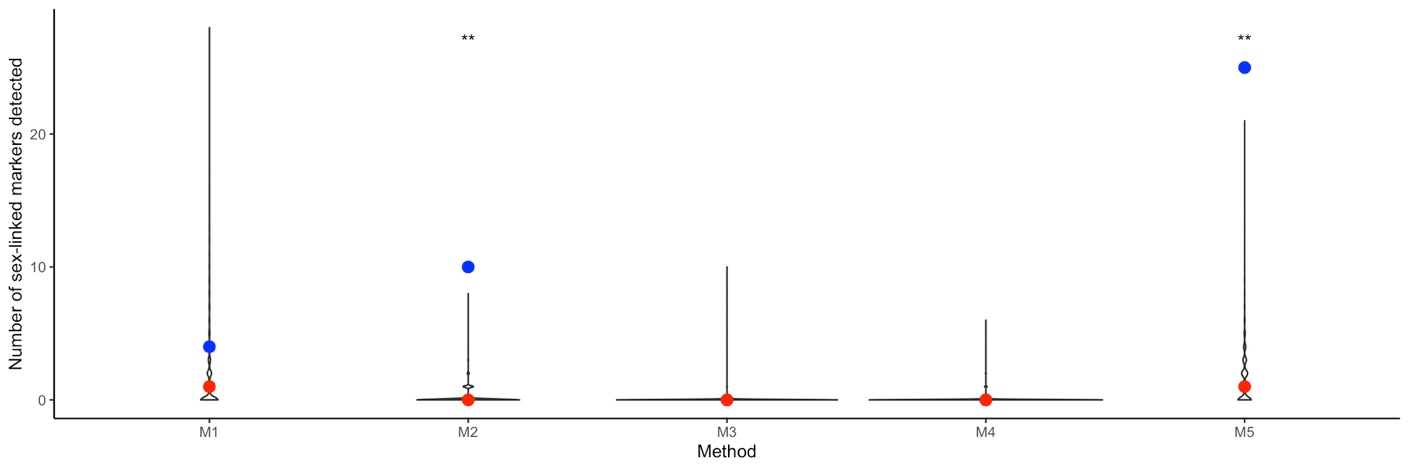


**Figure S9**: Violin plots of the number of markers identified during permutations for each of the five methods, with actual number of XY and ZW markers identified depicted in blue and red respectively. Method 2 and Method 5 are significant (p < 0.01).


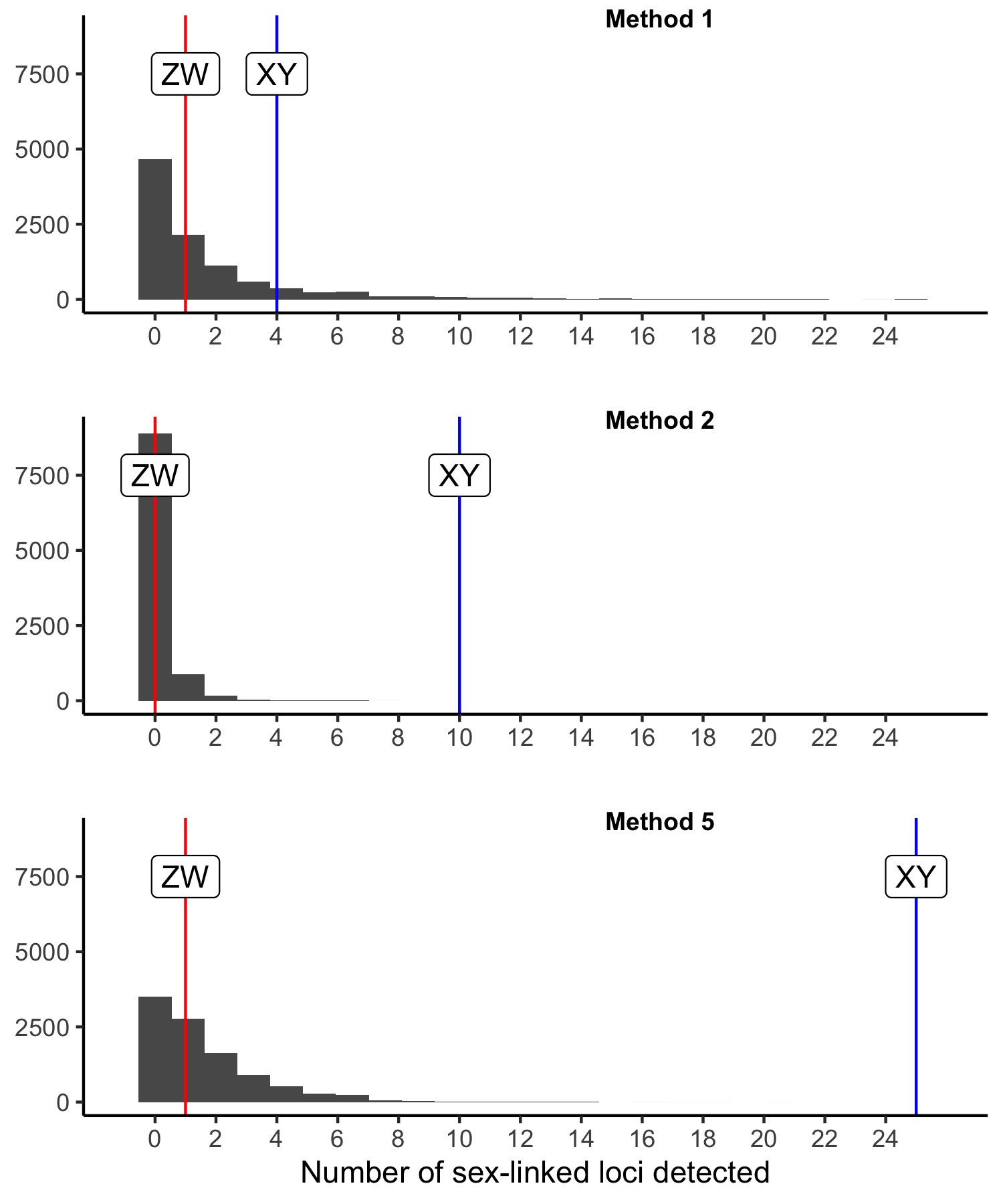


**Figure S10**: Frequency histograms showing the number of markers identified across permutations for Method 1, 2 and 5, with the actual number of XY (blue line) and ZW (red line) markers identified for each of the three methods overlayed. Method 2 and Method 5 are both significant (p < 0.01).

**Supplementary material 6**

**Details of sex-association analyses**

This section contains the QQplot for the sex-association analyses conducted in the SNPassoc package, and Manhattan plots before and after FDR correction.


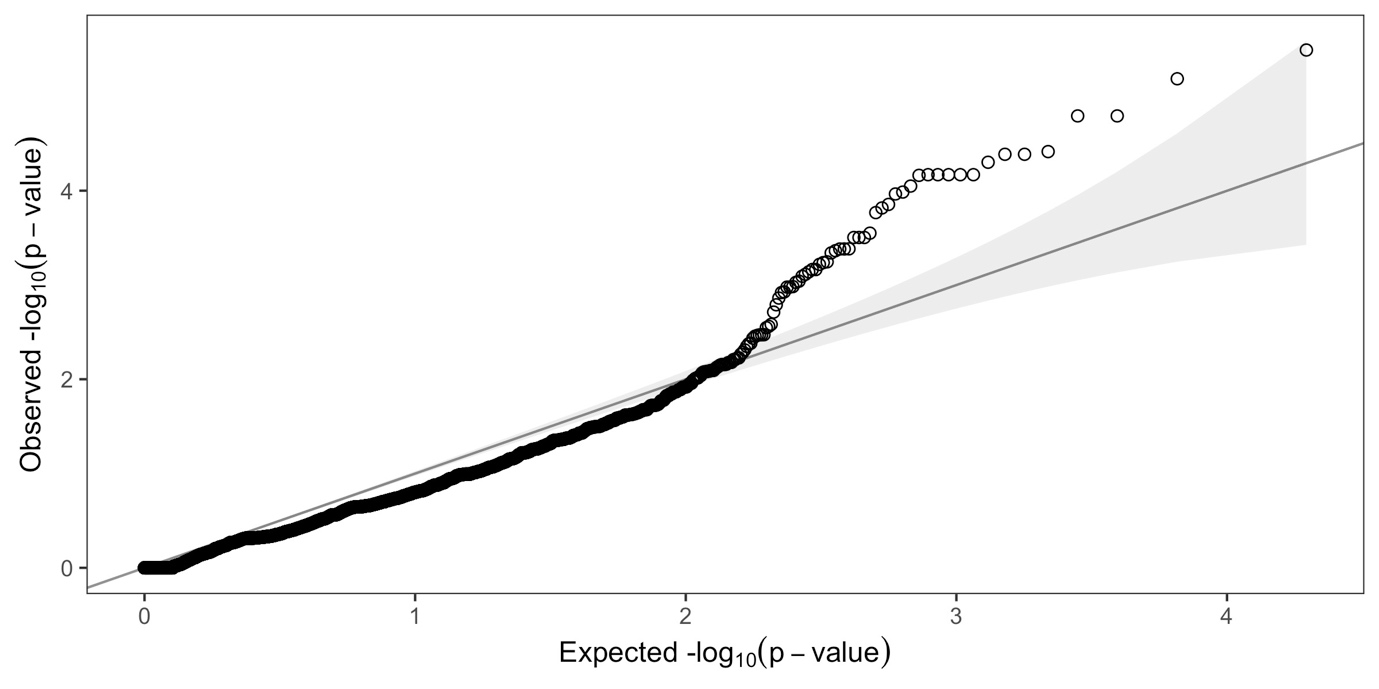


**Figure S11**: QQPlot of observed versus expected p-values (based on a uniform distribution), for all tested SNPs. To highlight smaller p-values all values are in the -log_10_ scale.


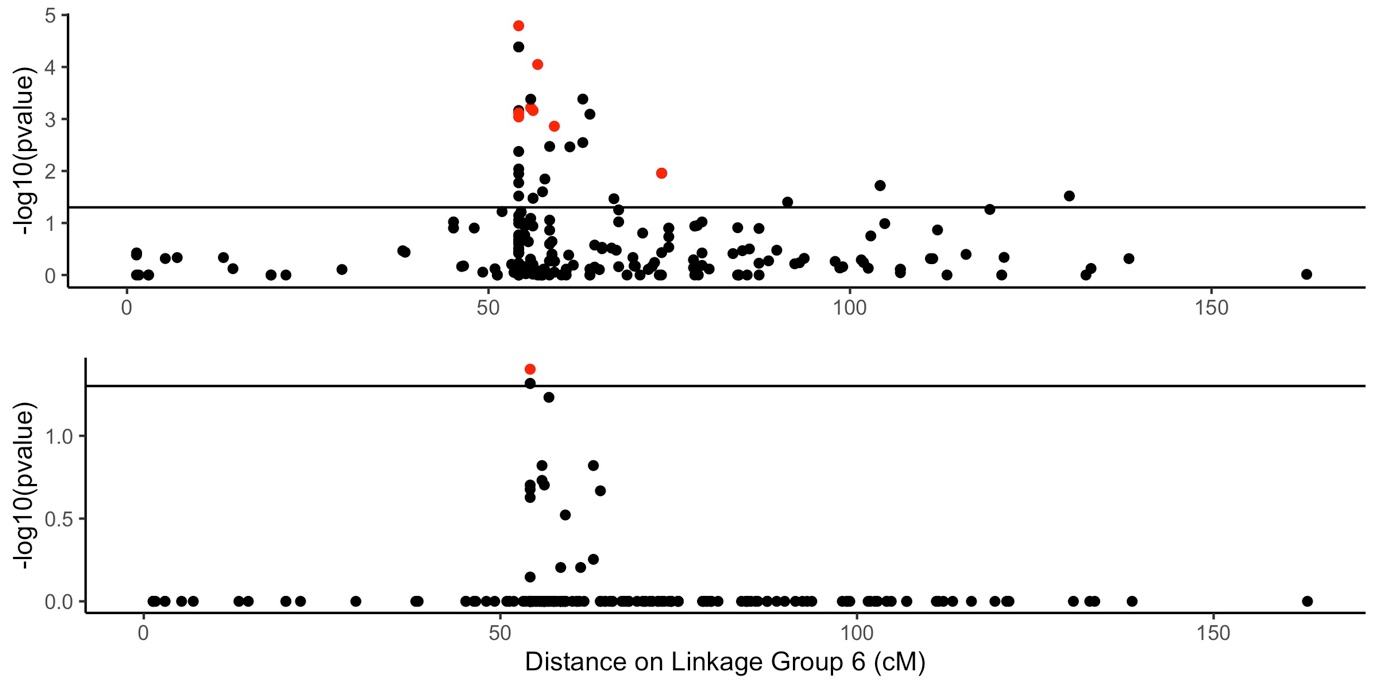


**Figure S12**: Manhattan plot of significance (-log_10_ scale) for individual SNP association tests for linkage group 6, before (top) and after (bottom) correcting for multiple testing with the FDR method. Markers depicted in red indicate significant sex-associated markers also identified by sex-linkage method.

**Supplementary material 7**

**Summary table of markers assessed in this study for the linkage mapping and sex determination analyses**

This file contains detailed information for all markers used in this study, including markers for linkage mapping and analyses of sex-linkage. For each marker, a markerID, fasta consensus sequence, linkage mapping parent genotypes, offspring genotype counts and proportions, adult sexed individuals genotype counts and proportions, and results from analyses of segregation distortion, sex-linkage, and sex-association are reported. Detailed metadata is included within the file.

Table S4: Summary table of genomic markers used in this study. Available separately from the Heredity website.

**Supplementary material 8**

**Summary of Blast results for linkage map markers**

In this section we provide a visual summary of the number of matches between the linkage map markers and two of the genome assemblies we mapped our RADtags to. Table s5 shows the *L. serrata* linkage groups as columns, and the *B. bufo/X. tropicalis* chromosome-level scaffold names as rows. Numbers represent the number of markers present in the corresponding linkage group the corresponding scaffold.

Table S5: Matches between *L. serrata* linkage map and the chromosome level assemblies of *Bufo bufo* (top) and *Xenopus tropicalis* (bottom)*.*

**Supplementary material 9**

**Summary of Blast results for sex markers**

Table S6: Table of blast results for RADtags containing sex-linked markers, including Locus ID in the stacks catalogue (as reported in Table S4), description of blast result and standard blast output details.


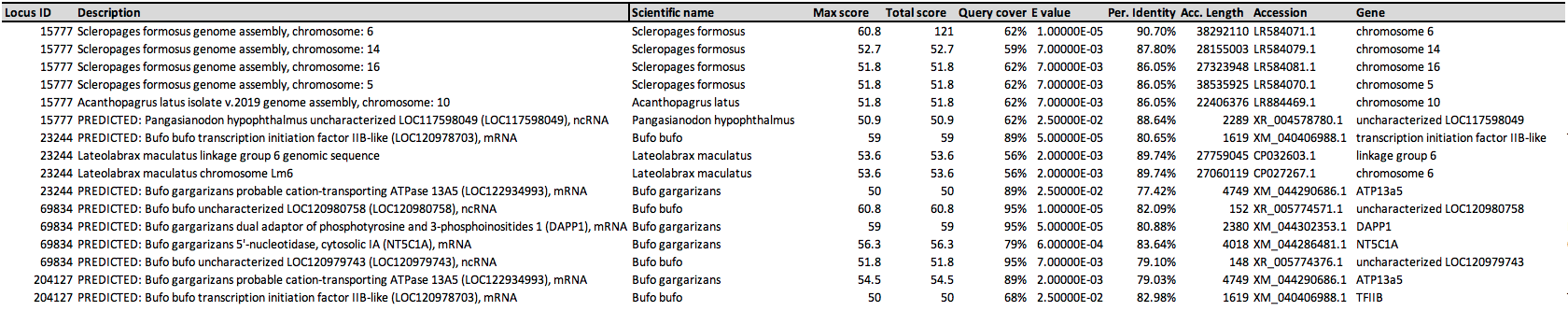

Supplement: Supplementary file 1 — Supplementary material [file 41437_2023_642_MOESM1_ESM.docx]
